# Using healthcare claims data to analyze the prevalence of BCR‐ABL‐positive chronic myeloid leukemia in France: A nationwide population‐based study

**DOI:** 10.1002/cam4.2200

**Published:** 2019-04-30

**Authors:** Stéphanie Foulon, Pascale Cony‐Makhoul, Agnès Guerci‐Bresler, Marc Delord, Eric Solary, Alain Monnereau, Julia Bonastre, Pascale Tubert‐Bitter

**Affiliations:** ^1^ Biostatistics Unit Gustave Roussy Villejuif France; ^2^ CESP Centre for Research in Epidemiology and Population Health, INSERM U1018, Paris‐Sud Univ Villejuif France; ^3^ B2PHI Biostatistics, Biomathematics, Pharmacoepidemiology and Infectious Diseases Inserm U1181, UVSQ, Paris Saclay Univ Villejuif France; ^4^ Service d'Hématologie CH Annecy Genevois Pringy France; ^5^ FiLMC Group, Institut Bergonié Bordeaux France; ^6^ Service d'Hématologie CHRU Brabois Vandoeuvre France; ^7^ Institut Universitaire d'Hématologie Université Paris‐Diderot Paris7 Paris France; ^8^ Department of Hematology Gustave Roussy Villejuif France; ^9^ INSERM U1170 Villejuif France; ^10^ University of Bordeaux, Inserm, Bordeaux Population Health Research Center, Team EPICENE, UMR 1219 Bordeaux France; ^11^ Registre des Hémopathies Malignes de la Gironde, Institut Bergonié Bordeaux France; ^12^ French Network of Population‐based Cancer Registries (FRANCIM) Toulouse France

**Keywords:** cancer registries, chronic myeloid leukemia, epidemiology, insurance claims database, prevalence

## Abstract

**Background:**

Data on Chronic Myeloid Leukemia (CML) prevalence are scarce. Here we provide an estimation of the prevalence of CML in France for the year 2014 using French national health insurance data.

**Methods:**

We selected patients claiming reimbursement for tyrosine kinase inhibitors (TKI) or with hospital discharge diagnoses for CML, BCR/ABL‐positive or with full reimbursement of health care expenses for myeloid leukemia. We built an algorithm which we validated on a random sample of 100 potential CML patients by comparing the results obtained using the algorithm and the opinion of two hematologists who reviewed the patient demographics and sequence of care abstracted from claims data (internal validity). For external validity, we compared the number of incident CML patients identified using the algorithm with those recorded in French population‐based cancer registries in departments covered by such a registry.

**Results:**

We identified 10 789 prevalent CML patients in 2014, corresponding to a crude prevalence rate of 16.3 per 100 000 inhabitants [95% confidence interval (CI) 16.0‐16.6]: 18.5 in men [18.0‐19.0] and 14.2 in women [13.8‐14.6]. The crude CML prevalence was less than 1.6 per 100 000 [1.2‐2.0] under age 20, increasing to a maximum of 48.2 [45.4‐51.2) at ages 75‐79. It varied from 10.2 to 23.8 per 100 000 across French departments. The algorithm showed high internal and external validity. Concordance rate between the algorithm and the hematologists was 96%, and the numbers of incident CML patients identified using the algorithm and the registries were 162 and 150, respectively.

**Conclusion:**

We built and validated an algorithm to identify CML patients in administrative healthcare databases. In addition to prevalence estimation, the algorithm could be used for future economic evaluations or pharmaco‐epidemiological studies in this population.

## 1 INTRODUCTION

Chronic Myeloid Leukemia (CML) is a myeloproliferative neoplasm whose age‐adjusted annual incidence rate is around 1 per 100 000 person‐years in European countries.^1^, ^2^ Since the introduction of tyrosine kinase inhibitors (TKI) in the early 2000s, the survival of patients with CML has improved dramatically.^3^, ^4^, ^5^, ^6^, ^7^ As CML incidence increases with age, an aging population combined with an improvement in survival is expected to increase CML prevalence. Based on the estimations from 22 cancer registries in Europe, the prevalence of CML was estimated to be 5.6 per 100 000 in 2008.^8^ In Sweden, CML prevalence was estimated at 5.7 per 100 000 in 2000 and 11 per 100 000 in 2012.^4^, ^9^ In the United States, the number of CML cases was estimated to be 70 000 in 2010, indicating a high prevalence of 22.6 per 100 000 inhabitants, with a predicted number of cases reaching 112 000 in 2020.^10^ In France, a regional hospital‐based evaluation of CML prevalence (Nord‐Pas de Calais region) indicated an increase in CML prevalence from 5.8 to 10.4 per 100 000 inhabitants between 1998 and 2007.^11^ In France, CML prevalence was recently modeled from 1960 to 2060 by using incidence rates from six French population‐based cancer registries (corresponding to 11 departments and covering 14% of the French population). Scenarios combining projections of the French population and various hypotheses on the evolution of relative survival of CML patients were proposed. In the base case scenario, CML prevalence was estimated at 2.5 per 100 000 inhabitants before the 1980s, 6.4 in 2002, 13.7 in 2012, and 17.5 in 2018, with an anticipated plateau at 32 per 100 000 inhabitants in the 2060s.^12^ These estimates of CML prevalence were obtained through a modeling exercise using data on relative survival from other European countries. Modeling estimates of CML prevalence were consistent with the number of imatinib sales in 2004 and the regional estimate of CML prevalence in Nord‐Pas de Calais until 2007. But since then, no recent data were available to validate modeling estimates.

Administrative healthcare databases are regularly used to assess the economic burden of diseases and to allocate resources both at the national and the regional level. More recently, these data sources have been extensively used in economic evaluation to estimate input parameters in cost‐effectiveness studies. Similarly, administrative and claims data are used to estimate target populations and market shares for pricing and reimbursement of new medicines as well as postmarketing surveillance studies. In addition, administrative databases are relevant sources of data to conduct epidemiological studies worldwide.^13^, ^14^, ^15^, ^16^, ^17^, ^18^ In the present study, our objective was to build and validate an algorithm to assess the prevalence of CML at the nationwide level, using individual data from French national insurance databases. Our aim was also to make available an algorithm to identify CML patients in administrative healthcare databases that could be used for future economic evaluations or pharmaco‐epidemiological studies in this patient population.

## 2 PATIENTS AND METHODS

### 2.1 Study design and data sources

We performed a cross‐sectional study using data from the French national health insurance database linked with the national hospital discharge database.^19^, ^20^, ^21^ These databases contain individualized, anonymous, and comprehensive data on all healthcare reimbursements (including hospital stays coded with the International Classification of Diseases, 10th Revision (ICD‐10) diagnosis codes, medications, general practitioner and specialist consultations, imaging and biological procedures and sick/disability leaves) and patient eligibility for full reimbursement of health care expenses related to specific costly or long‐term diseases (LTD) also coded with ICD‐10 codes. Claims data are gathered across nearly all French Health Insurance schemes, covering 98.8% of the population living in France (66.4 million inhabitants on 1 January 2015). Besides healthcare reimbursement data, demographic data are also available including the year of birth, gender, area of residence, and date of death. There was no requirement for ethics approval to be sought for this observational study, based on anonymous healthcare claims data.

### 2.2 Algorithm to identify CML patients

#### 2.2.1 Selection of potential CML patients

For the 2006‐2014 period, we selected from the French national health insurance databases: (a) all patients treated by TKI indicated in CML (imatinib, dasatinib, nilotinib, bosutinib or ponatinib) and/or (b) identified by the ICD‐10 diagnosis code C92.1 (Chronic Myeloid Leukemia, BCR/ABL‐positive) among hospital discharge diagnoses and/or (c) having requested full reimbursement of health care expenses for Myeloid Leukemia (ICD‐10 diagnosis code C92). The latter criterion (ICD‐10 diagnosis code C92 for Myeloid Leukemia) is coded only with three characters in the French national health insurance databases. It is therefore not specific for Chronic Myeloid Leukemia and patients with a very different disease such as Acute Myeloid Leukemia could also be selected using this ICD‐10 code. However, at the time of the data extraction, we preferred not to be too restrictive in our selection criteria. Then, to refine this selection, we examined the sequence of care between 2006 and 2014 for 200 randomly selected individuals, using a standardized form (Figure S1). For each individual, this form presented demographic data and healthcare resource utilization.

#### 2.2.2 Algorithm building

Based on this thorough examination and clinical opinions, we built a claim‐based algorithm to identify CML patients. Case definition was based on (a) identifying any TKI reimbursement associated with a hospitalization for CML or lasting ≥2 months and (b) excluding patients receiving TKIs for diseases other than CML including Philadelphia positive Acute Lymphoblastic Leukemia, Gastrointestinal Stromal Tumor, Graft versus Host Disease, Hyper Eosinophilic Syndrome and Stromal or other Connective Tissue Tumor. These differential diagnoses were identified using discharge diagnoses, Long‐Term Disease diagnoses and reimbursement of specific drugs (Figure S2). The SAS program of the algorithm is available upon request from the corresponding author.

### 2.3 CML prevalence

Prevalent CML cases were those identified using the algorithm described above, having at least one healthcare reimbursement during the year 2014 and still alive on 31 December 2014. The nationwide crude CML prevalence rate was defined as the number of prevalent CML patients divided by the number of people living in France on 1 January 2015 (www.insee.fr) 22 and is provided with an exact Clopper‐Pearson confidence interval.^23^ The national CML prevalence rate was also standardized by gender and by 5‐year age group to the 1976 European population, and the 1960 World population, to allow for international comparisons.^24^ Both crude and standardized rates correspond to estimates of the complete prevalence of CML. Crude and standardized prevalence rates by department, using the department of residence of prevalent CML patients in 2014, were computed to explore geographical variations in CML prevalence in the French territory. The reference population for the standardization on gender and age was the general population residing in France on 1 January 2015.

### 2.4 Algorithm validity

The internal validity of our algorithm was assessed from another random sample of 100 potential CML patients. We calculated the proportion of agreement between the result of the algorithm and the opinion of two expert hematologists from the FiLMC group. For each individual, hematologists reviewed the standardized patient form presenting patient demographics and sequence of care from claims data.

In addition, we performed an external validation of our algorithm using data from French cancer registries. We compared the number of incident CML cases (ie new CML cases) identified from the French national health insurance databases for 3 years from 2012 to 2014 with the number of incident CML cases (ICD‐O‐3 9875/3: Chronic myelogenous leukemia, BCR/ABL‐positive) recorded in 18 French population‐based cancer registries for the same years and the same geographical areas (covering 22% of the French population). In the French national health insurance databases, the index date for the diagnosis of CML was defined as the first date among the first date of TKI or hydroxycarbamide reimbursement, or the start date for full reimbursement of health care expenses for myeloid leukemia or the first date of hospitalization for CML. In a sensitivity analysis, the CML prevalence rate was adjusted using the results of external validation (cf. Supplementary). Statistical analyses were performed using SAS 9.3.

## 3 RESULTS

### 3.1 Population study

**Figure 1:**
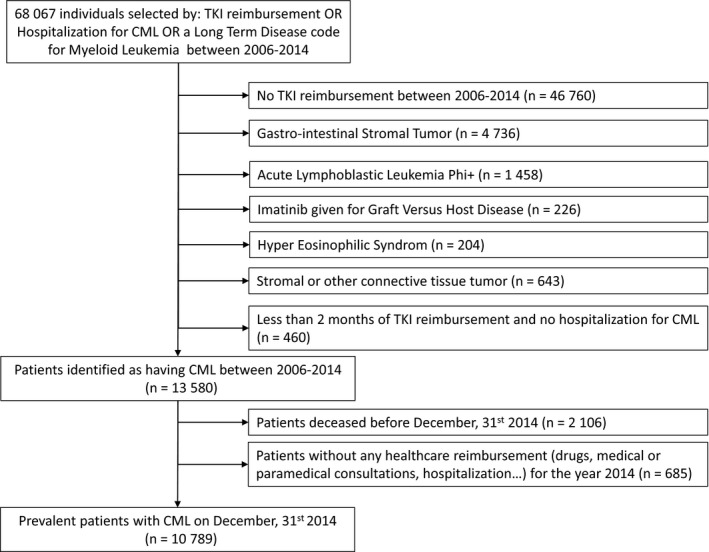
Patient flow chart. Data were extracted from the French national health insurance databases for the period 2006‐2014. TKI: Tyrosine Kinase Inhibitor, CML: Chronic Myeloid Leukemia

68 067 individuals were selected from the French national health insurance databases using the selection criteria described in the methods section (paragraph 2.2.1). Applying the algorithm (paragraph 2.2.2) resulted in the identification of 10 789 patients with CML on 31 December 2014 (Figure 1). Forty‐eight percent of these prevalent CML cases had the three selection criteria (TKI, hospitalization with a CML diagnosis code, full reimbursement of health care expenses for myeloid leukemia), 32% were treated by TKI and benefited from full reimbursement of health care expenses for myeloid leukemia, 9% had TKI and hospitalization with CML diagnosis code. Finally, 11% of the CML patients identified using the algorithm were captured by TKI reimbursement only. The median age [interquartile range] of the prevalent population of CML patients was 63 [51‐73], with a slight male preponderance (55%).

### 3.2 Estimation of CML prevalence

**Figure 2:**
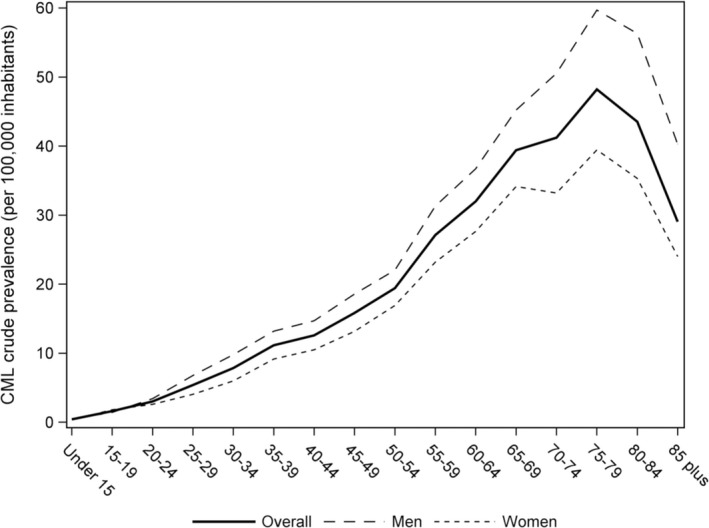
Age‐specific and gender‐specific crude CML prevalence rates in France in 2014. This figure represents the CML crude prevalence rate by age and sex of the population prevalent on 31 December 2014

**Figure 3:**
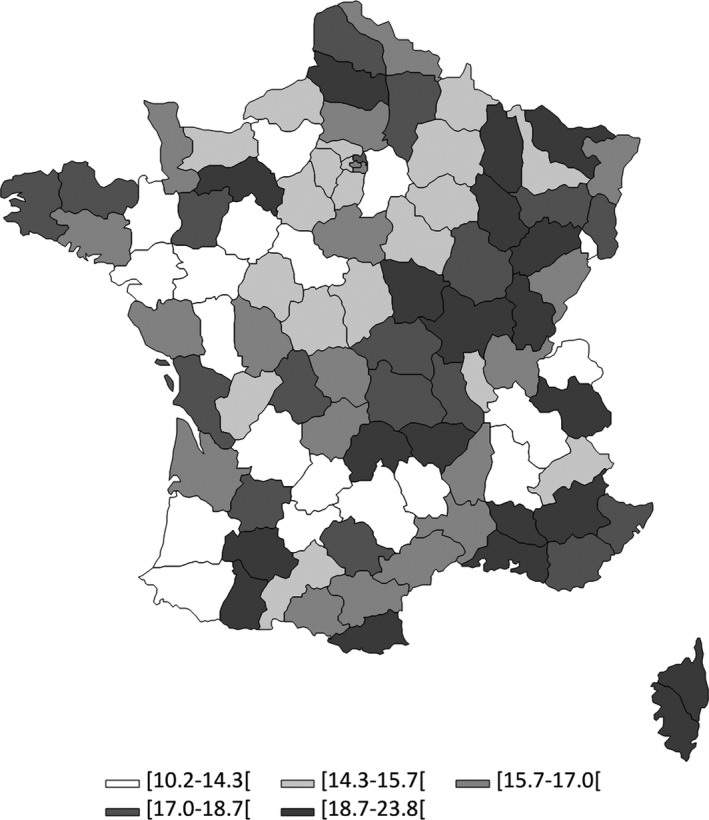
CML prevalence rates in 2014 by Department in France. CML: Chronic Myeloid Leukemia. These are crude estimates of CML prevalence per 100 000 inhabitants. Departments are those in which prevalent CML patients lived in 2014

On 31 December 2014, the crude prevalence of CML was estimated at 16.3 per 100 000 inhabitants in France [95% confidence interval (CI) 16.0‐16.6]. The crude prevalence of CML was 18.5 per 100 000 in men [95% CI 18.0‐19.0] and 14.2 per 100,000 in women [95% CI 13.8‐14.6]. To allow for international comparisons, standardized prevalence rates are shown in Table 1. CML prevalence increased with age (Figure 2 and Table S2). The crude prevalence of CML was less than 1.6 per 100 000 [95% CI 1.2‐2.0] before 20, progressively increased to 19.4 [95% CI 18.1‐20.8] at 50‐54 years and reached a peak of 48.2 [95% CI 45.4‐51.2] at 75‐79 years. There was a preponderance of CML in men in all age groups except in younger age groups where prevalence was comparable between genders. The crude prevalence of CML varied from 10.2 to 23.8 per 100 000 inhabitants across all French departments (Figure 3). These variations of prevalence persisted to a lesser extent after standardization on gender and age (Table S1).

**Table 1:** Crude and standardized prevalence rates of CML in France, in 2014

	Overall	Men	Women
Number of prevalent CML patients on 31 December 2014	10 789	5931	4858
French population in 2014	66 226 643	32 076 965	34 149 678
Crude prevalence of CML per 100 000 inhabitants	16.3 [16.0‐16.6]	18.5 [18.0‐19.0]	14.2 [13.8‐14.6]
Standardized prevalence rates per 100 000 (1960‐World population)	10.0 [9.8‐10.3]	11.7 [11.4‐12.0]	8.4 [8.1‐8.6]
Standardized prevalence rates per 100 000 (1976‐European population)	13.3 [13.1‐13.6]	15.6 [15.2‐16.0]	11.1 [10.8‐11.4]

Abbreviation: CML: Chronic Myeloid Leukemia.

### 3.3 Algorithm validation to identify CML patients

The comparison of the algorithm with the opinion of hematologists indicated a 96% concordance rate (N = 100). According to the hematologists, the algorithm correctly identified 45 true CML patients and correctly excluded 51 false CML patients. However, the algorithm missed three patients who had less than 2 months of TKI reimbursement but were considered as CML patients by the hematologists, based on their sequence of care. Finally, the algorithm selected one false positive CML patient. This was a patient with reimbursement of imatinib for a short time period and missing data for both hospitalization and eligibility status to full reimbursement of health care expenses for myeloid leukemia.

For the year 2014, the 18 population‐based cancer registries from the Francim network identified 150 incident CML patients whereas the algorithm identified 162 new CML patients for the same areas. The median age [interquartile range] of the incident population of CML patients identified using the algorithm was 60 [46‐71], with a slight male preponderance (57%). Considering the cancer registries as the gold standard, the algorithm overestimated the number of incident CML patients by 8% in 2014 compared to cancer registries. In 2013 and 2012, the algorithm overestimated the number of incident CML patients by 13% and 14% respectively. In a sensitivity analysis, we provide estimates adjusted using the results of the external validation. The adjusted crude CML prevalence rate was estimated at 15.1 per 100 000 [95% CI 14.8‐15.4] in 2014:17.1 per 100 000 in men [95% CI 16.7‐17.6] and 13.2 per 100 000 in women [95% CI 12.8‐13.5].

## 4 DISCUSSION

Based on the French national health insurance databases, we estimated that the crude prevalence of CML in France was 16.3 per 100 000 inhabitants in 2014. The male preponderance, reported in incidence studies,^2^, ^8^, ^25^, ^26^ persisted when studying prevalence and was constant across the ages, except in younger age groups where prevalence was comparable between genders.

Using different sources of data and methodology, our results are consistent with the estimates from Delord et al which ranged from 13.7 per 100 000 inhabitants in 2012 to 17.5 in 2018.^12^ CML prevalence estimated by Delord et al was obtained from projections of the French population, incidence rates from six French population‐based cancer registries studies and hypotheses on the relative survival of CML patients, while we used individual data from the French national health insurance databases. Our results in 2014 are also consistent with the rising trend in the CML prevalence estimated by Corm et al in the French region Nord‐Pas de Calais, from 5.8 per 100 000 inhabitants in 1997 to 10.4 in 2007.^11^ Our CML prevalence estimate of 16.3 per 100 000 inhabitants in France in 2014 is between the Swedish estimate (11 per 100 000 inhabitants in 2012 and 14 per 100 000 in 2018)^4^, ^9^ and the US prevalence estimates (22.6 per 100 000 in 2010 and 33.5 per 100 000 in 2020).^10^ These differences may reflect discrepancies in age distribution, leading to different incidence of CML but also differences in access to TKI with an impact on survival, and differences in methods. It is worth noting that the difference in CML prevalence between Sweden and United States is mainly related to quite different incidence rates (0.90 per 100 000 in Sweden and 1.75 per 100 000 in the United States), which may be due to a wider definition of CML used in the US SEER register or to real differences between the two countries. The increase in CML prevalence may lead physicians to modify their practices in order to care for an increasing number of patients and consider new modalities of follow‐up such as clinical routine assessment by clinical nurses. Physicians will also have to adapt to the aging of this population, with a peak of prevalence at 75‐79 years in our study, as elderly patients may be more sensitive to side effects of second generation TKIs.

There were variations in CML prevalence across the French territory, which cannot be only explained by differences in age and gender distribution of the population in the French departments (cf. crude and standardized CML prevalence by department Table S1). Data presented here correspond to the distribution of the prevalent CML patients in the departments in which they lived in 2014. These variations are unlikely to be explained by a different quality of data reporting across the departments because information on reimbursement of TKI, on which the algorithm mainly relies, is collected the same way across the French territory. Data regarding environmental exposures are not available in the National Health Insurance databases, precluding association studies using individual data for exposure and outcomes. However, our finding opens research prospects for geographical and ecologic studies.

The French national health insurance databases used in the present study have several advantages: (a) data are collected prospectively and are readily available to be analyzed, (b) the databases cover 98.8% of French population, hence avoiding selection bias and providing a large sample size, (c) data are available from 2006 onwards with no losses to follow‐up. A key issue in studies using healthcare administrative databases is to develop and validate an algorithm that identifies patients with the highest accuracy. Different initiatives exist worldwide to encourage the building, validation and use of these algorithms, such as the review of algorithms used to detect various outcomes of interest within the Mini‐Sentinel program in the US,^27^ and the REDSIAM network in France.^28^ Building such algorithms requires expertise in both the studied disease and the administrative databases used.

The analysis of the sequence of care of 200 randomly selected individuals by two expert hematologists using a standardized form was a major step in the building of the algorithm. Indeed, hematologists found a certain amount of miscoding of hematological diagnoses for hospital stays. For example, for a given patient, some hospital discharges were coded CML while it was obvious that the patient suffered from Chronic MyeloMonocytic Leukemia (CMML), another hematological malignancy, given the sequence of care of the patients (treatments, other hospital discharge coded CMML, prognosis…). For that reason, we based our algorithm on the reimbursement of TKIs rather than on hospitalizations with a CML diagnosis code.

To our knowledge, the rare previous studies on CML using healthcare administrative databases^29^, ^30^, ^31^ chose TKI reimbursement combined with claims associated with diagnosis for CML (ICD‐9 code: 205.1) to analyze patterns of TKI treatment and healthcare resources consumption without an attempt to quantify prevalence.

Our work has several strengths. Firstly, our algorithm was validated internally by two expert hematologists and externally by comparison with the number of incident CML patients recorded in 18 French population‐based cancer registries in the same geographical areas. Secondly, our estimate of CML prevalence is based on individual data at the nationwide level. It also provides estimates of CML prevalence in different geographical areas across France using an identical method of measurement. It can allow comparisons and improve health policy decision‐making. Finally, our algorithm could be used to reestimate the CML prevalence rate in France periodically. The algorithm could also be used or adapted in other countries to identify CML patients, provided those countries have databases containing reimbursement/dispensation of drugs data linked with medical discharge diagnosis databases.

Our study also has limitations. The first limitation was the frequent miscoding of hematological diagnoses for hospital stays, which explains why we based our algorithm on the reimbursement of TKIs rather than on hospitalizations with a CML diagnosis code. Hence, the algorithm is unable to identify three categories of true CML patients: (a) patients who have always been treated by interferon alone, (b) patients who have always received TKI in a clinical trial setting, the TKI being provided by the sponsor and thus not reimbursed by the National Health Insurance and (c) elderly patients who have been treated by cytoreductive chemotherapy only. The proportion of patients treated by interferon alone was estimated at 0.7% in a cross‐sectional study on the management of first line CML patients in 2013 in France.^32^ Regarding the patients who received TKIs in the setting of clinical trials, searching the EU Clinical Trials Register, we found 25 clinical trials starting between 2004 and 2014 in France in CML patients. Excluding trials in which the patients had to be previously treated by TKI to be included in the trial, we estimated an upper range of 628 patients that could have been missed by the algorithm. Among those patients, several discontinued the trials and were thus identifiable by the algorithm if treated by a TKI outside the trial. In our internal validation, we identified three patients out of 100 who had not received TKI but were considered by the hematologists as possible CML patients given their whole sequences of care. These patients could correspond to the three different mentioned situations. Therefore, the impact of these limitations on the estimation of CML prevalence should be limited.

Secondly, we were unable to validate CML patients identified with our algorithm using medical records because data from the French national health insurance databases for research are anonymous. This difficulty was partially overcome through internal and external validations. However, for the same reason of anonymity, we were unable to perform an individual matching with the registries but we were able to compare total numbers of incident cases between the two sources of data. The algorithm slightly overestimated the number of incident CML patients (from 14% in 2012 to 8% in 2014). Non‐CML patients receiving TKI for others diseases than the differential diagnoses already excluded by the algorithm may lead to this slight overestimation. As a sensitivity analysis, we provided adjusted CML prevalence estimates taking into account this possible overestimation. Lastly, the algorithm depends on the care pathway of CML in general and more specifically on the indications of prescription of TKI at the time when we built the algorithm. In the future, some patients in deep molecular remissions may opt for a controlled discontinuation of TKI therapy. They will no longer be treated (and thus identifiable) by TKI. However, before entering into a treatment discontinuation strategy, patients must be treated by TKI for at least 5 years^33^ and are therefore identifiable by their TKI intake during this period. To continue to use the algorithm in the coming years, the observation period should be extended as much as possible and start before 2007, year of beginning of inclusion in the first French clinical trial (STIM1) assessing controlled discontinuation of TKI therapy. If new TKIs were to be commercialized, the algorithm should be adapted to take into account this change, by adding new drugs in the list of TKIs.

In conclusion, we built and validated an algorithm to identify CML patients in healthcare administrative databases. We estimated the crude CML prevalence in France at 16.3 per 100 000 inhabitants in 2014, using an innovative methodology based on data from the French national health insurance databases. The algorithm could enable periodical re‐estimation of the CML prevalence rate in France and could be implemented in other countries using healthcare claims databases. The algorithm will also offer the opportunity to study healthcare pathways and healthcare uses of French CML patients or to study the association between adverse events and CML treatments in the French national health insurance databases.

## CONFLICT OF INTEREST

The authors indicate no potential conflicts of interest.

## DATA AVAILABILITY STATEMENT

The data underlying the findings cannot be made freely available because of legal restrictions. Data used for the present study come from the French National Health Insurance databases and include an important number of variables, that, when combined the ones to the others, can lead to re‐idendifying subjects, and then collecting health information on these persons. This is why the French Data Protection Authority (CNIL) forbids us to make such data freely available. Access to the raw data of the French National Health Insurance must be requested from the National Institute of Health Data (https://www.indsante.fr/).
