# Supplementary material for: Using healthcare claims data to analyze the prevalence of BCR‐ABL‐positive chronic myeloid leukemia in France: A nationwide population‐based study
Source: Cancer Med. 2019 Apr 30;8(6):3296–304. doi: 10.1002/cam4.2200 (PMC6558491; doi:10.1002/cam4.2200)
Supplement: Supplementary file 1 [file CAM4-8-3296-s001.docx]

**SUPPLEMENTARY**

**Method to estimate an adjusted crude prevalence (sensitivity analysis)**

To account for possible overestimation of the number of incident CML patients using the algorithm, we estimated an adjusted crude prevalence as follows.

Using the external validation study (comparison between the number of incident patients identified in the French health insurance database versus the registries), we estimate a relative error E on the number of the incident patients:

$$E=\frac{{N incident cases}_{French health insurance database}-{N incident cases}_{Registries}}{{N incident cases}_{Registries}}$$

Under the assumptions that the population of patients is initially in steady state and that the frequency of the disease is rare, the prevalence of CML can be considered proportional to the incidence of CML. Thus, we can correct our estimate of the prevalence of CML from the French health insurance database by dividing this estimate by a factor (1+ relative error E).

**Table S1. Crude and standardized CML prevalence rates in 2014 in each French department (except Mayotte and overseas territories)**

| Department | Number of CML patients | Number of people living in the department | Crude prevalence (per 100,000 inhabitants) | 95% confidence interval | Standardized prevalence per 100,000 inhabitants) | 95% confidence interval |
| --- | --- | --- | --- | --- | --- | --- |
| **TOTAL** | **10,789** | **66,226,643** | **16.3** | **[ 16.0- 16.6]** | **16.3** | **[ 16.0 - 16.6 ]** |
| 01-AIN | 100 | 633,588 | 15.8 | [ 12.8- 19.2] | 16.6 | [ 13.4 - 19.9 ] |
| 02-AISNE | 94 | 539,058 | 17.4 | [ 14.1- 21.3] | 17.0 | [ 13.5 - 20.4 ] |
| 03-ALLIER | 63 | 343,266 | 18.4 | [ 14.1- 23.5] | 15.7 | [ 11.8 - 19.7 ] |
| 04-ALPES-DE-HAUTE-PROVENCE | 38 | 161,619 | 23.5 | [ 16.6- 32.3] | 19.0 | [ 12.9 - 25.2 ] |
| 05-HAUTES-ALPES | 20 | 140,225 | 14.3 | [ 8.7- 22.0] | 12.9 | [ 7.2 - 18.5 ] |
| 06-ALPES-MARITIMES | 199 | 1,083,952 | 18.4 | [ 15.9- 21.1] | 16.5 | [ 14.2 - 18.9 ] |
| 07-ARDECHE | 51 | 324,014 | 15.7 | [ 11.7- 20.7] | 14.5 | [ 10.5 - 18.6 ] |
| 08-ARDENNES | 41 | 278,503 | 14.7 | [ 10.6- 20.0] | 14.1 | [ 9.8 - 18.5 ] |
| 09-ARIEGE | 26 | 152,621 | 17.0 | [ 11.1- 25.0] | 15.5 | [ 9.4 - 21.6 ] |
| 10-AUBE | 46 | 309,294 | 14.9 | [ 10.9- 19.8] | 14.3 | [ 10.1 - 18.4 ] |
| 11-AUDE | 61 | 367,142 | 16.6 | [ 12.7- 21.3] | 14.4 | [ 10.8 - 18.1 ] |
| 12-AVEYRON | 37 | 279,672 | 13.2 | [ 9.3- 18.2] | 11.3 | [ 7.5 - 15.0 ] |
| 13-BOUCHES-DU-RHONE | 416 | 2,016,345 | 20.6 | [ 18.7- 22.7] | 20.3 | [ 18.4 - 22.3 ] |
| 14-CALVADOS | 100 | 693,500 | 14.4 | [ 11.7- 17.5] | 14.1 | [ 11.3 - 16.8 ] |
| 15-CANTAL | 33 | 146,268 | 22.6 | [ 15.5- 31.7] | 18.9 | [ 12.3 - 25.5 ] |
| 16-CHARENTE | 54 | 354,312 | 15.2 | [ 11.4- 19.9] | 13.4 | [ 9.8 - 17.0 ] |
| 17-CHARENTE-MARITIME | 116 | 640,828 | 18.1 | [ 15.0- 21.7] | 15.3 | [ 12.5 - 18.1 ] |
| 18-CHER | 48 | 309,814 | 15.5 | [ 11.4- 20.5] | 13.4 | [ 9.6 - 17.3 ] |
| 19-CORREZE | 41 | 241,002 | 17.0 | [ 12.2- 23.1] | 13.8 | [ 9.5 - 18.1 ] |
| 21-COTE-D'OR | 95 | 533,325 | 17.8 | [ 14.4- 21.8] | 17.7 | [ 14.2 - 21.3 ] |
| 22-COTES-D'ARMOR | 111 | 598,187 | 18.6 | [ 15.3- 22.3] | 15.9 | [ 12.9 - 18.9 ] |
| 23-CREUSE | 19 | 119,900 | 15.8 | [ 9.5- 24.7] | 12.2 | [ 6.7 - 17.8 ] |
| 24-DORDOGNE | 45 | 416,557 | 10.8 | [ 7.9- 14.5] | 9.0 | [ 6.3 - 11.8 ] |
| 25-DOUBS | 86 | 536,374 | 16.0 | [ 12.8- 19.8] | 16.4 | [ 12.9 - 19.9 ] |
| 26-DROME | 69 | 502,823 | 13.7 | [ 10.7- 17.4] | 12.9 | [ 9.8 - 15.9 ] |
| 27-EURE | 83 | 601,327 | 13.8 | [ 11.0- 17.1] | 14.3 | [ 11.2 - 17.4 ] |
| 28-EURE-ET-LOIR | 64 | 434,887 | 14.7 | [ 11.3- 18.8] | 14.5 | [ 11.0 - 18.1 ] |
| 29-FINISTERE | 159 | 907,747 | 17.5 | [ 14.9- 20.5] | 16.2 | [ 13.7 - 18.7 ] |
| 30-GARD | 122 | 741,706 | 16.4 | [ 13.7- 19.6] | 15.1 | [ 12.4 - 17.8 ] |
| 31-HAUTE-GARONNE | 191 | 1,337,098 | 14.3 | [ 12.3- 16.5] | 15.6 | [ 13.4 - 17.8 ] |
| 32-GERS | 37 | 191,212 | 19.4 | [ 13.6- 26.7] | 16.1 | [ 10.8 - 21.4 ] |
| 33-GIRONDE | 245 | 1,546,862 | 15.8 | [ 13.9- 18.0] | 16.1 | [ 14.1 - 18.1 ] |
| 34-HERAULT | 188 | 1,122,447 | 16.7 | [ 14.4- 19.3] | 16.3 | [ 13.9 - 18.6 ] |
| 35-ILLE-ET-VILAINE | 138 | 1,043,701 | 13.2 | [ 11.1- 15.6] | 14.1 | [ 11.7 - 16.4 ] |
| 36-INDRE | 35 | 224,760 | 15.6 | [ 10.8- 21.7] | 13.0 | [ 8.6 - 17.3 ] |
| 37-INDRE-ET-LOIRE | 88 | 607,390 | 14.5 | [ 11.6- 17.8] | 13.9 | [ 11.0 - 16.8 ] |
| 38-ISERE | 174 | 1,252,953 | 13.9 | [ 11.9- 16.1] | 14.3 | [ 12.2 - 16.5 ] |
| 39-JURA | 54 | 260,463 | 20.7 | [ 15.6- 27.1] | 19.3 | [ 14.1 - 24.5 ] |
| 40-LANDES | 56 | 404,353 | 13.8 | [ 10.5- 18.0] | 12.1 | [ 8.9 - 15.2 ] |
| 41-LOIR-ET-CHER | 45 | 334,202 | 13.5 | [ 9.8- 18.0] | 12.6 | [ 8.8 - 16.3 ] |
| 42-LOIRE | 138 | 759,987 | 18.2 | [ 15.3- 21.5] | 17.2 | [ 14.3 - 20.1 ] |
| 43-HAUTE-LOIRE | 49 | 227,104 | 21.6 | [ 16.0- 28.5] | 19.7 | [ 14.0 - 25.3 ] |
| 44-LOIRE-ATLANTIQUE | 185 | 1,363,233 | 13.6 | [ 11.7- 15.7] | 14.2 | [ 12.2 - 16.3 ] |
| 45-LOIRET | 111 | 672,652 | 16.5 | [ 13.6- 19.9] | 16.3 | [ 13.3 - 19.4 ] |
| 46-LOT | 24 | 173,197 | 13.9 | [ 8.9- 20.6] | 10.8 | [ 6.4 - 15.2 ] |
| 47-LOT-ET-GARONNE | 60 | 333,905 | 18.0 | [ 13.7- 23.1] | 14.8 | [ 10.9 - 18.6 ] |
| 48-LOZERE | 10 | 76,093 | 13.1 | [ 6.3- 24.2] | 12.2 | [ 4.5 - 19.8 ] |
| 49-MAINE-ET-LOIRE | 110 | 810,763 | 13.6 | [ 11.2- 16.4] | 13.9 | [ 11.3 - 16.5 ] |
| 50-MANCHE | 79 | 499,878 | 15.8 | [ 12.5- 19.7] | 14.1 | [ 10.9 - 17.2 ] |
| 51-MARNE | 84 | 572,280 | 14.7 | [ 11.7- 18.2] | 15.1 | [ 11.9 - 18.3 ] |
| 52-HAUTE-MARNE | 36 | 180,079 | 20.0 | [ 14.0- 27.7] | 17.5 | [ 11.8 - 23.3 ] |
| 53-MAYENNE | 55 | 307,218 | 17.9 | [ 13.5- 23.3] | 17.2 | [ 12.6 - 21.7 ] |
| 54-MEURTHE-ET-MOSELLE | 112 | 731,741 | 15.3 | [ 12.6- 18.4] | 15.3 | [ 12.5 - 18.2 ] |
| 55-MEUSE | 38 | 190,741 | 19.9 | [ 14.1- 27.3] | 18.7 | [ 12.7 - 24.6 ] |
| 56-MORBIHAN | 119 | 745,510 | 16.0 | [ 13.2- 19.1] | 14.6 | [ 12.0 - 17.3 ] |
| 57-MOSELLE | 203 | 1,045,043 | 19.4 | [ 16.8- 22.3] | 19.1 | [ 16.4 - 21.7 ] |
| 58-NIEVRE | 47 | 211,920 | 22.2 | [ 16.3- 29.5] | 19.0 | [ 13.4 - 24.6 ] |
| 59-NORD | 433 | 2,611,596 | 16.6 | [ 15.1- 18.2] | 18.2 | [ 16.5 - 19.9 ] |
| 60-OISE | 137 | 823,020 | 16.6 | [ 14.0- 19.7] | 17.7 | [ 14.7 - 20.7 ] |
| 61-ORNE | 56 | 286,524 | 19.5 | [ 14.8- 25.4] | 17.3 | [ 12.7 - 21.8 ] |
| 62-PAS-DE-CALAIS | 262 | 1,475,944 | 17.8 | [ 15.7- 20.0] | 18.6 | [ 16.3 - 20.8 ] |
| 63-PUY-DE-DOME | 114 | 647,318 | 17.6 | [ 14.5- 21.2] | 16.8 | [ 13.7 - 19.9 ] |
| 64-PYRENEES-ATLANTIQUES | 84 | 670,816 | 12.5 | [ 10.0- 15.5] | 11.4 | [ 8.9 - 13.8 ] |
| 65-HAUTES-PYRENEES | 44 | 228,900 | 19.2 | [ 14.0- 25.8] | 16.5 | [ 11.5 - 21.4 ] |
| 66-PYRENEES-ORIENTALES | 112 | 470,830 | 23.8 | [ 19.6- 28.6] | 21.0 | [ 17.1 - 24.9 ] |
| 67-BAS-RHIN | 185 | 1,117,503 | 16.6 | [ 14.3- 19.1] | 16.9 | [ 14.5 - 19.4 ] |
| 68-HAUT-RHIN | 141 | 762,480 | 18.5 | [ 15.6- 21.8] | 18.2 | [ 15.2 - 21.3 ] |
| 69-RHONE | 281 | 1,821,898 | 15.4 | [ 13.7- 17.3] | 17.4 | [ 15.3 - 19.4 ] |
| 70-HAUTE-SAONE | 56 | 237,772 | 23.6 | [ 17.8- 30.6] | 21.3 | [ 15.7 - 26.9 ] |
| 71-SAONE-ET-LOIRE | 119 | 555,695 | 21.4 | [ 17.7- 25.6] | 19.3 | [ 15.8 - 22.8 ] |
| 72-SARTHE | 71 | 569,816 | 12.5 | [ 9.7- 15.7] | 11.9 | [ 9.2 - 14.7 ] |
| 73-SAVOIE | 87 | 429,604 | 20.3 | [ 16.2- 25.0] | 19.7 | [ 15.6 - 23.8 ] |
| 74-HAUTE-SAVOIE | 104 | 795,273 | 13.1 | [ 10.7- 15.8] | 13.8 | [ 11.1 - 16.5 ] |
| 75-PARIS | 371 | 2,210,849 | 16.8 | [ 15.1- 18.6] | 18.2 | [ 16.3 - 20.1 ] |
| 76-SEINE-MARITIME | 188 | 1,259,488 | 14.9 | [ 12.9- 17.2] | 15.1 | [ 12.9 - 17.2 ] |
| 77-SEINE-ET-MARNE | 198 | 1,391,091 | 14.2 | [ 12.3- 16.4] | 16.3 | [ 14.0 - 18.6 ] |
| 78-YVELINES | 205 | 1,424,121 | 14.4 | [ 12.5- 16.5] | 15.7 | [ 13.5 - 17.9 ] |
| 79-DEUX-SEVRES | 50 | 374,307 | 13.4 | [ 9.9- 17.6] | 12.5 | [ 9.0 - 16.0 ] |
| 80-SOMME | 116 | 571,542 | 20.3 | [ 16.8- 24.3] | 20.5 | [ 16.8 - 24.2 ] |
| 81-TARN | 66 | 386,655 | 17.1 | [ 13.2- 21.7] | 15.0 | [ 11.3 - 18.6 ] |
| 82-TARN-ET-GARONNE | 26 | 255,118 | 10.2 | [ 6.7- 14.9] | 9.8 | [ 6.0 - 13.6 ] |
| 83-VAR | 195 | 1,046,733 | 18.6 | [ 16.1- 21.4] | 15.9 | [ 13.6 - 18.2 ] |
| 84-VAUCLUSE | 104 | 556,932 | 18.7 | [ 15.3- 22.6] | 17.6 | [ 14.2 - 21.0 ] |
| 85-VENDEE | 106 | 668,480 | 15.9 | [ 13.0- 19.2] | 14.6 | [ 11.8 - 17.4 ] |
| 86-VIENNE | 70 | 434,906 | 16.1 | [ 12.5- 20.3] | 15.3 | [ 11.7 - 18.8 ] |
| 87-HAUTE-VIENNE | 69 | 376,276 | 18.3 | [ 14.3- 23.2] | 16.6 | [ 12.7 - 20.6 ] |
| 88-VOSGES | 64 | 371,649 | 17.2 | [ 13.3- 22.0] | 15.3 | [ 11.6 - 19.1 ] |
| 89-YONNE | 50 | 341,533 | 14.6 | [ 10.9- 19.3] | 13.5 | [ 9.7 - 17.2 ] |
| 90-TERRITOIRE DE BELFORT | 18 | 144,630 | 12.4 | [ 7.4- 19.7] | 12.8 | [ 6.9 - 18.7 ] |
| 91-ESSONNE | 192 | 1,282,995 | 15.0 | [ 12.9- 17.2] | 16.8 | [ 14.4 - 19.2 ] |
| 92-HAUTS-DE-SEINE | 234 | 1,603,272 | 14.6 | [ 12.8- 16.6] | 15.9 | [ 13.8 - 18.0 ] |
| 93-SEINE-ST-DENIS | 276 | 1,585,800 | 17.4 | [ 15.4- 19.6] | 21.0 | [ 18.4 - 23.6 ] |
| 94-VAL-DE-MARNE | 223 | 1,376,442 | 16.2 | [ 14.1- 18.5] | 18.5 | [ 16.1 - 21.0 ] |
| 95-VAL-D'OISE | 186 | 1,214,125 | 15.3 | [ 13.2- 17.7] | 17.8 | [ 15.2 - 20.4 ] |
| CORSE | 62 | 327,374 | 18.9 | [ 14.5- 24.3] | 17.1 | [ 12.8 - 21.4 ] |
| DOM | 284 | 1,882,695 | 15.1 | [ 13.4- 16.9] | 17.8 | [ 15.7 - 20.0 ] |

**Table S2. Age-specific and gender-specific crude CML prevalence rates in France in 2014**

|  |  |  | Overall | | |  | | Men | | |  | |  | | Women | | |
| --- | --- | --- | --- | --- | --- | --- | --- | --- | --- | --- | --- | --- | --- | --- | --- | --- | --- |
| Age | Number of CML patients | Number of people living in France | CML crude prevalence | 95% confidence interval |  | | CML crude prevalence | | 95% confidence interval |  | |  | | CML crude prevalence | | 95% confidence interval |  |
| **TOTAL FRANCE** | **10,789** | **66,226,643** | **16.3** | **[ 16.0- 16.6]** |  | | **18.5** | | **[ 18.0- 19.0]** |  | |  | | **14.2** | | **[ 13.8- 14.6]** |  |
| Under 15 | 51 | 12,251,358 | 0.4 | [ 0.3- 0.5] |  | | 0.5 | | [ 0.3- 0.7] |  | |  | | 0.4 | | [ 0.2- 0.6] |  |
| 15-19 | 64 | 3,987,312 | 1.6 | [ 1.2- 2.0] |  | | 1.4 | | [ 1.0- 2.0] |  | |  | | 1.8 | | [ 1.3- 2.5] |  |
| 20-24 | 115 | 3,824,506 | 3.0 | [ 2.5- 3.6] |  | | 3.4 | | [ 2.6- 4.3] |  | |  | | 2.6 | | [ 1.9- 3.4] |  |
| 25-29 | 213 | 3,953,478 | 5.4 | [ 4.7- 6.2] |  | | 6.8 | | [ 5.7- 8.0] |  | |  | | 4.0 | | [ 3.2- 5.0] |  |
| 30-34 | 324 | 4,128,722 | 7.8 | [ 7.0- 8.8] |  | | 9.8 | | [ 8.5- 11.2] |  | |  | | 6.0 | | [ 5.0- 7.1] |  |
| 35-39 | 450 | 4,038,158 | 11.1 | [ 10.1- 12.2] |  | | 13.2 | | [ 11.6- 14.9] |  | |  | | 9.2 | | [ 7.9- 10.6] |  |
| 40-44 | 572 | 4,547,102 | 12.6 | [ 11.6- 13.7] |  | | 14.7 | | [ 13.2- 16.4] |  | |  | | 10.5 | | [ 9.2- 11.9] |  |
| 45-49 | 712 | 4,498,496 | 15.8 | [ 14.7- 17.0] |  | | 18.6 | | [ 16.8- 20.4] |  | |  | | 13.2 | | [ 11.7- 14.7] |  |
| 50-54 | 867 | 4,465,418 | 19.4 | [ 18.1- 20.8] |  | | 22.0 | | [ 20.1- 24.1] |  | |  | | 16.9 | | [ 15.3- 18.7] |  |
| 55-59 | 1,147 | 4,230,564 | 27.1 | [ 25.6- 28.7] |  | | 31.3 | | [ 28.9- 33.8] |  | |  | | 23.2 | | [ 21.2- 25.3] |  |
| 60-64 | 1,305 | 4,079,804 | 32.0 | [ 30.3- 33.8] |  | | 36.7 | | [ 34.1- 39.5] |  | |  | | 27.6 | | [ 25.4- 30.0] |  |
| 65-69 | 1,461 | 3,708,350 | 39.4 | [ 37.4- 41.5] |  | | 45.2 | | [ 42.1- 48.5] |  | |  | | 34.1 | | [ 31.6- 36.8] |  |
| 70-74 | 1,007 | 2,443,316 | 41.2 | [ 38.7- 43.8] |  | | 50.5 | | [ 46.5- 54.9] |  | |  | | 33.2 | | [ 30.2- 36.5] |  |
| 75-79 | 1,069 | 2,217,322 | 48.2 | [ 45.4- 51.2] |  | | 59.7 | | [ 54.9- 64.8] |  | |  | | 39.4 | | [ 36.0- 43.1] |  |
| 80-84 | 824 | 1,893,167 | 43.5 | [ 40.6- 46.6] |  | | 56.3 | | [ 51.0- 62.0] |  | |  | | 35.3 | | [ 32.0- 38.9] |  |
| 85 plus | 569 | 1,959,570 | 29.0 | [ 26.7- 31.5] |  | | 40.3 | | [ 35.4- 45.7] |  | |  | | 24.0 | | [ 21.5- 26.8] |  |

Figure S1: Example of a form for a fictitious patient

**Patient n° XXXXX**

**Demographic characteristics**

**Sex:** Male

**Year of birth:** 1942

**Age on 1^st^ January 2006:** 64 years

**Month and year of death :** .

**Social security scheme:** General scheme

**Long Term Diseases**

| **Long Term Disease** | **Date of start of full reimbursement of expenses** | **Date of end of the of full reimbursement of expenses** |
| --- | --- | --- |
| Myeloid Leukemia | 20/03/2012 | 12/04/2018 |

**Hospitalizations between 2006 and 2014**

| **Hospitalization** | **Principal diagnosis** | **Related diagnosis** | **Associated diagnoses** | **Start date of hospitalization** | **Length of hospital stay (in days)** |
| --- | --- | --- | --- | --- | --- |
| **2012 – Hospitalization n°1** | Other specified disorders of white blood cells |  | Osteoarthritis of knee, unspecified \| Venous insufficiency (chronic) (peripheral) \| Pure hypercholesterolemia \| Other specified hypothyroidism\| Essential (primary) hypertension | 30/06/2012 | 3 |
| **2012 – Hospitalization n°2** | Chronic myeloid leukemia, BCR/ABL-positive |  | Other specified hypothyroidism \| Other specified disorders of white blood cells \| Essential (primary) hypertension | 30/08/2012 | 10 |

**Outpatient treatment (ATC codes of molecules related to onco-hematology)**

| **Name of the delivered medicine** | **Year** | **Jan** | **Feb** | **Mar** | **Apr** | **May** | **Jun** | **Jul** | **Aug** | **Sep** | **Oct** | **Nov** | **Dec** |
| --- | --- | --- | --- | --- | --- | --- | --- | --- | --- | --- | --- | --- | --- |
| GLIVEC 400MG CPR 30 | 2012 | 0 | 0 | 0 | 0 | 0 | 0 | 0 | 0 | 1 | 1 | 1 | 1 |
| GLIVEC 400MG CPR 30 | 2013 | 1 | 1 | 1 | 1 | 1 | 1 | 1 | 1 | 1 | 2 | 0 | 1 |
| GLIVEC 400MG CPR 30 | 2014 | 1 | 1 | 1 | 1 | 1 | 0 | 1 | 1 | 1 | 1 | 2 | 0 |

**Medical consultations (with a hematologist, an oncologist, a gastroenterologist or an internal medicine specialist)**

| **Medical specialty of the consulting physician** | **Year** | **Jan** | **Feb** | **Mar** | **Apr** | **May** | **Jun** | **Jul** | **Aug** | **Sep** | **Oct** | **Nov** | **Dec** |
| --- | --- | --- | --- | --- | --- | --- | --- | --- | --- | --- | --- | --- | --- |
| INTERNAL MEDICINE | 2012 | 0 | 0 | 0 | 0 | 0 | 0 | 0 | 1 | 0 | 0 | 0 | 0 |
| HEMATOLOGY | 2012 | 0 | 0 | 0 | 0 | 0 | 0 | 0 | 0 | 1 | 1 | 1 | 0 |
| HEMATOLOGY | 2013 | 1 | 1 | 0 | 0 | 1 | 0 | 1 | 0 | 0 | 0 | 0 | 0 |
| HEMATOLOGY | 2014 | 0 | 1 | 0 | 0 | 0 | 0 | 1 | 0 | 0 | 0 | 0 | 0 |

**Figure S2. Description of the patients selected with the selection criteria and building of the algorithm**


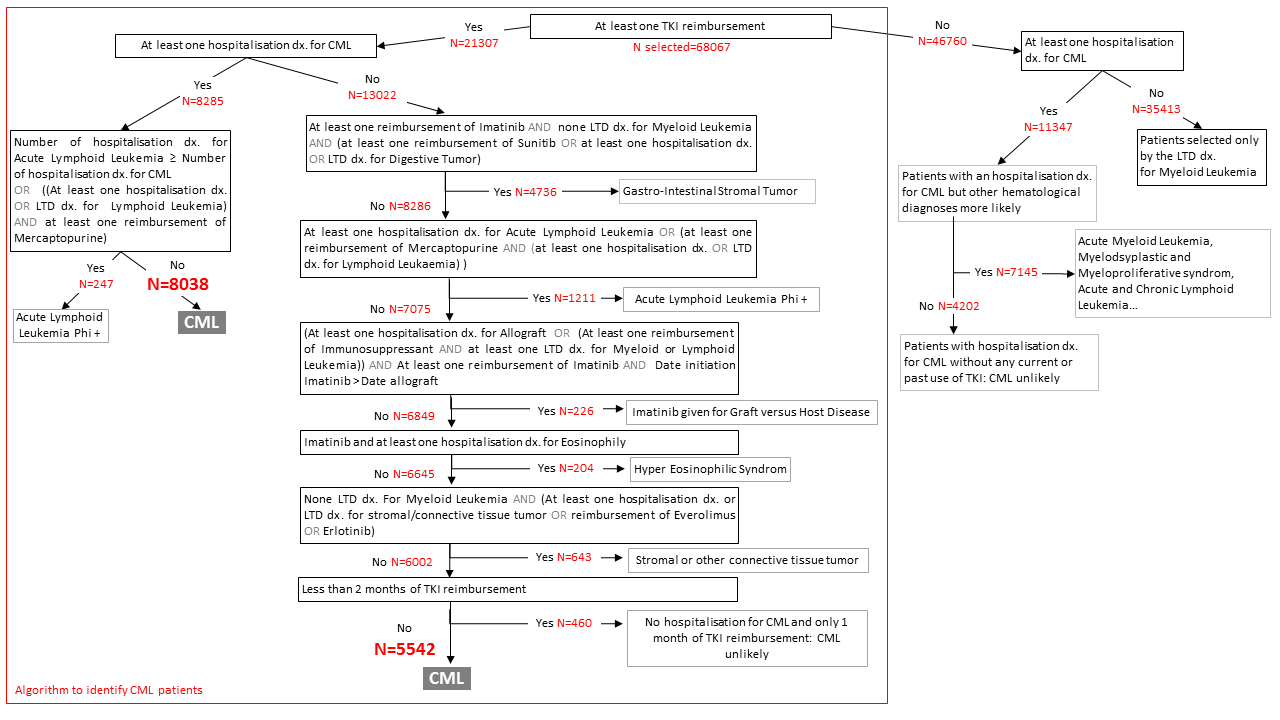


Dx.: Diagnosis, CML: Chronic Myeloid Leukemia, TKI: Tyrosine Kinase Inhibitor, LTD: Long Term Disease (full reimbursement of health care expenses)

**ICD10 codes used for:**

- Acute Lymphoid Leukemia (C910)
- Allograft : Allograft (Z94801) OR Bone marrow transplant rejection (T8601) OR Bone marrow transplant failure (T8602)
- Chronic Myeloid Leukemia (C921)
- Digestive Tumor:

Malignant neoplasm of oesophagus (C15x)* OR Malignant neoplasm of stomach (C16x) OR Malignant neoplasm of small intestine (C17x) OR Malignant neoplasm of colon (C18x) OR Malignant neoplasm of rectosigmoid junction (C19x) OR Malignant neoplasm of rectum (C20x) OR Malignant neoplasm of anus and anal canal (C21x) OR Malignant neoplasm of liver and intrahepatic bile ducts (C22x) OR Malignant neoplasm of gallbladder (C23x) OR Malignant neoplasm of other and unspecified parts of biliary tract (C24x) OR Malignant neoplasm of pancreas (C25x) OR Malignant neoplasm of other and ill-defined digestive organs (C26x) OR Malignant neoplasm of retroperitoneum and peritoneum (C48x) OR Neoplasm of uncertain or unknown behaviour of oral cavity and digestive organs (D37x), , Neoplasm of uncertain or unknown behaviour of other and unspecified sites, peritoneum (D484),

- Eosinophily : Chronic eosinophilic leukemia [hypereosinophilic syndrome] (D475) OR Eosinophilia (D721)
- Graft Versus Host Disease: Bone marrow transplant rejection (T8601) OR Bone marrow transplant failure (T8602)
- Lymphoid Leukemia (C91x)
- Myeloid Leukemia (C92x)
- Stromal/connective tissue tumor:

Malignant neoplasm of bone and articular cartilage of limbs (C40), Malignant neoplasm of bone and articular cartilage of other and unspecified sites (C41), Other malignant neoplasms of skin (C44), Malignant neoplasm of other connective and soft tissue (C49) , Secondary malignant neoplasm of skin (C792 ), Myeloid sarcoma(C923), Neoplasm of uncertain or unknown behaviour of other and unspecified sites, connective and other soft tissue (D481) , Neoplasm of uncertain or unknown behaviour of other and unspecified sites, bone and articular cartilage (D480), Neoplasm of uncertain or unknown behaviour of other and unspecified sites, retroperitoneum(D483), Neoplasm of uncertain or unknown behaviour of other and unspecified sites skin (D485), Personal history of Malignant neoplasm of bone and articular cartilage (Z85803)

* C15x means that the ICD codes can be C15 or C150 or C151 or C152…

**ATC codes of drugs:**

TKI indicated for the treatment of CML:

- Imatinib: L01XE01
- Dasatinib: L01XE06
- Nilotinib: L01XE08
- Bosutinib: L01XE14
- Ponatinib: L01XE24

Other treatments:

- Sunitinib: L01XE04
- Immunosuppressants :
  - Ciclosporin: L04AD01
  - Mycophenolic acid: L04AA06
  - Sirolimus: L04AA10
  - Tacrolimus: D11AH01
- Everolimus: L01XE10
- Erlotinib: L01XE03
- Mercaptopurine: L01BB02
